# Acute changes in saliva metabolomic profile after drinking water instead of apple juice or no drink signal improved insulin-related metabolism in healthy children: a randomized controlled trial

**DOI:** 10.3389/fnut.2026.1812940

**Published:** 2026-10-22

**Authors:** Jodi D. Stookey, Mimansa Cholera, Rowena Cape

**Affiliations:** 1 Epidemiology Unit, Maternal, Child, & Adolescent Health, San Francisco Department of Public Health, San Francisco, CA, United States; 2 Department of Orofacial Sciences Pediatric Dentistry, University of California San Francisco, San Francisco, CA, United States

**Keywords:** children, drinking water, juice, metabolomics, randomized trial, saliva insulin

## Abstract

**Objective:**

In the Saliva Insulin as Biomarker (SIB) randomized controlled trial (RCT), saliva insulin increased more, within 60 min, after one serving of apple juice, and normalized less, after no drink, compared to the acute change after one serving of drinking water. This paper tested for corresponding drink treatment effects on metabolomic profile, recognizing that effects on insulin, a metabolic switch, imply co-occurring effects on many metabolic pathways, simultaneously, and potential for school-based drinking water interventions to efficiently target risk factor profiles. Metabolic pathways potentially impacted by each drink treatment were described by baseline weight condition to generate hypotheses about potential metabolic targets for drinking water intervention.

**Methods:**

Untargeted metabolomic assays were completed for saliva from 105 healthy children (5–10y), who participated in the 3-arm parallel group SIB RCT (NCT05211843), prior to a routine dental check-up on a weekday morning or afternoon. Unstimulated saliva was collected, at baseline, before study participants were randomly assigned to drink 500 mL water (W), 200 mL apple juice (AJ), or no drink (ND), and 45–60 min post-drink treatment. Randomization and analysis were stratified by age-and-sex-specific BMI percentile (5–85th or >85th). Drink treatment effects on change in saliva metabolomic profiles were compared using orthogonal partial least squares discriminant analysis (OPLS-DA), permutational multivariate analysis of variance (PERMANOVA), and Metaboanalyst 6.0 pathway module.

**Results:**

W significantly altered the saliva metabolomic profile compared with AJ in children with normal weight (validated OPLS-DA, Q^2^ = 0.30, permutation *p* = 0.005; PERMANOVA *p* = 0.04) and compared with ND in children with overweight or obesity (Q2 = 0.29; permutation *p* = 0.04; PERMANOVA *p* = 0.02). Increases or decreases of 1.5-fold or more in over 100 metabolites with KEGG IDs, after W instead of either AJ or ND, potentially impacted dozens of metabolic pathways.

**Conclusion:**

In healthy children, with normal weight, overweight or obesity, W instead of AJ or ND caused acute changes in saliva metabolomic profile that signal potential for multiple weight- and drink treatment-specific metabolic targets for drinking water intervention. The results warrant further research to pursue potential metabolic benefits of drinking water.

**Clinical trial registration:**

NCT05211843, clinicaltrials.gov

## 1 Introduction

Health authorities recommend school-based drinking water intervention to efficiently prevent childhood obesity and chronic disease risk (1–3). Obesity affects an estimated 188 million children worldwide (4). Obesity in childhood significantly increases risk of adult diabetes, cardiovascular disease, and some cancers (5). Beyond expectation that drinking water intervention could limit obesity by lowering total energy intake, consistently observed effects of drinking water on *insulin* (6), imply additional potential for a *wide range* of *metabolic benefits* of drinking water intervention. To warrant drinking water interventions to pursue metabolic outcomes in children, this randomized controlled trial (RCT) reports changes in metabolomic profile after one serving of drinking water, instead of one serving of apple juice, or no drink, in elementary school-aged children. Aside from potential to reduce chronic disease incidence and/or progression, interventions that target early metabolic precursors of chronic disease offer opportunity for shorter intervention follow-up, facilitating faster Plan-Do-Study-Act (PDSA) cycles and intervention quality improvement.

School-based drinking water interventions must address beverage exposures that are routinely available to children during the school day. Data to tailor interventions to the available beverage options, are lacking, however. Health authority guidance about drinking water for chronic disease prevention [e.g., (7, 8)] does not include detail about *how much* plain water to drink, if it should be consumed *alone or instead of* other fluid sources, the *purpose* of drinking water to reduce specific target health risk factors, and *what magnitude of effect* to expect in children, given the setting. Systematic reviews over the past 20 years have repeatedly concluded that evidence is insufficient to inform drinking water recommendations for chronic disease prevention (9–16). Despite expectation that school-based water interventions can efficiently promote healthy weight for millions of children around the world, 83 of 115 school-based drinking water interventions report null effects (6). Many null effect interventions are not specified to align with conditions for causal effects of drinking water (17). Data from RCTs about causal effects of drinking water exposures, which are relevant for school-based intervention, could inform choice of specific beverage change goals and specific metabolic outcomes to target for school-based drinking water interventions.

Drinking water may have absolute and/or relative effects on insulin and metabolic outcomes. Drinking water effects may, furthermore, depend on baseline weight status. Drinking more vs. less water, in absolute terms, can be hypothesized to impact metabolism via hydration-mediated mechanisms, by reducing hyperosmotic stress, which can reduce insulin resistance (18–21). Drinking water may be hypothesized to impact insulin in relative terms, if consumed instead of a caloric beverage, by reducing macronutrient-stimulated insulin secretion. Weight status can be expected to modify the metabolic pathways impacted and/or magnitudes of effect, because overweight and obesity are associated with significantly elevated insulin levels and metabolic dysregulation (22–25). Failure to account for baseline weight status is one of the possible reasons for intervention null effects (6, 17, 26).

To address gaps in the RCT evidence base for drinking water, this paper reports effects of the Saliva Insulin as Biomarker (SIB) RCT on saliva metabolomic profile. The SIB RCT tested for absolute and/or relative effects of drinking water exposures that are routinely available at schools in San Francisco. One standard serving of drinking water (500 mL bottle), instead of one serving of apple juice (200 mL), or no drink, significantly reduced the relative odds of elevated saliva insulin, within 1 h (27). The present analysis weight-stratified the SIB RCT insulin effect and checked for corresponding drink treatment effects on change in saliva metabolomic profile.

The goal of the SIB RCT was to generate data that can inform drinking water intervention planning. The SIB RCT was motivated by a high local prevalence of caries experience in kindergartners (27%) (28), a high local prevalence of child overweight or obesity (35% of 5th graders with a body mass index above the Fitnessgram ‘Healthy Fitness Zone’ (29)), and local investment in school-based drinking water interventions (30). The SIB RCT evaluated *saliva* insulin and metabolomics, instead of blood parameters, because saliva is a non-invasive, painless measure, which is feasible for use in non-clinical, school-based settings, facilitates group-representative, generalizable data collection, provides a near-linear correlation with fasting blood insulin (31), and enables early-stage cardiometabolic risk screening (32). The specific aims of this secondary-outcome analysis were to (1) check for potential effect modification by baseline weight, (2) test for randomized drink treatment effects on overall change in saliva metabolomic profile, and (3) provide descriptive data to generate hypotheses about specific metabolic pathways potentially impacted by drinking water intervention in children with normal weight and children with overweight or obesity, respectively.

## 2 Methods

### 2.1 Study design and protocol

The methods of this weight-status stratified, parallel group, randomized controlled trial (RCT) have been described, in detail, with the primary results of the SIB RCT (27). All data collection happened at one pediatric dental clinic in San Francisco in 2022–2023. For each study participant, study enrollment, baseline data collection, randomization, and follow-up data collection happened on the same day, within 1 h. Study participants were measured for weight status classification on arrival at the clinic, consented for study participation in the Normal weight or Overweight or Obesity group, and randomly assigned to have one of three weight-specific drink treatments: Water (W), Apple juice (AJ), or No drink (ND), immediately after baseline data collection. The drink treatment was administered immediately after randomization, after which time the study participants remained sedentary, completing the study questionnaire, in the same room with their parent or caregiver, for 1 h. Follow-up saliva was collected at 45–60 min after the drink treatment. Each study participant received $40 for completing the protocol and proceeded to their scheduled dental service. The protocol was reviewed and approved by the University of California San Francisco, Human Research Protection Program Institutional Review Board (#21–35,500) and registered at clinicaltrials.gov (NCT05211843). The study participants and staff who collected the study specimen were not blind to the intervention treatment. Laboratory and epidemiologist staff who analyzed the saliva and data were blind to the treatment allocation.

### 2.2 Participant recruitment

Study participants were recruited via a large community-wide referral and health record (EPIC) system and roster for one pediatric dental clinic. All pediatric patients, ages 5–10 years, who were already scheduled or due to have a new or recall dental exam, were invited to participate. One dental clinician (MC) handled all recruitment outreach, pre-screening, and participant scheduling. Children were excluded from study participation if they were unable to speak English, Spanish, or Chinese, not living in a county eligible for San Francisco school-based services, coming to the dental clinic for an operative procedure, not healthy enough to attend school (fever or systemic conditions), unable or unwilling to drink water or apple juice, or unable or unwilling to give saliva. For convenience, study participation was scheduled for the hour immediately before the scheduled preventive dental service, either in the morning or afternoon. Study staff weighed and measured each child on arrival at the clinic, and enrolled children into weight-specific groups based on age-and-sex-specific BMI percentile (5–85: normal weight; >85: overweight or obesity) and CDC cutoffs (33). Written parental consent and child assent were obtained.

The CONSORT flow diagram in Figure 1 describes the number of children who were recruited, consented, randomized, administered a drink treatment, and followed for outcome measures, by child weight status. The original enrollment goal, of 60 children with normal weight and 60 children with overweight or obesity, based on power calculations to test RCT effects on the primary outcome (27), was not met. Dental clinic staff-time ran out. One participant with overweight or obesity, who was randomly assigned to drink apple juice, declined to drink the juice and dropped out after randomization. Missing data for the 45–60 min timepoint, for the one participant who dropped out, were replaced with the average value for participants with overweight or obesity, with similar baseline, followed after no drink. The present analysis included data from 105 healthy children, including 60 children with normal weight and 45 children with overweight or obesity.

**Figure 1:**
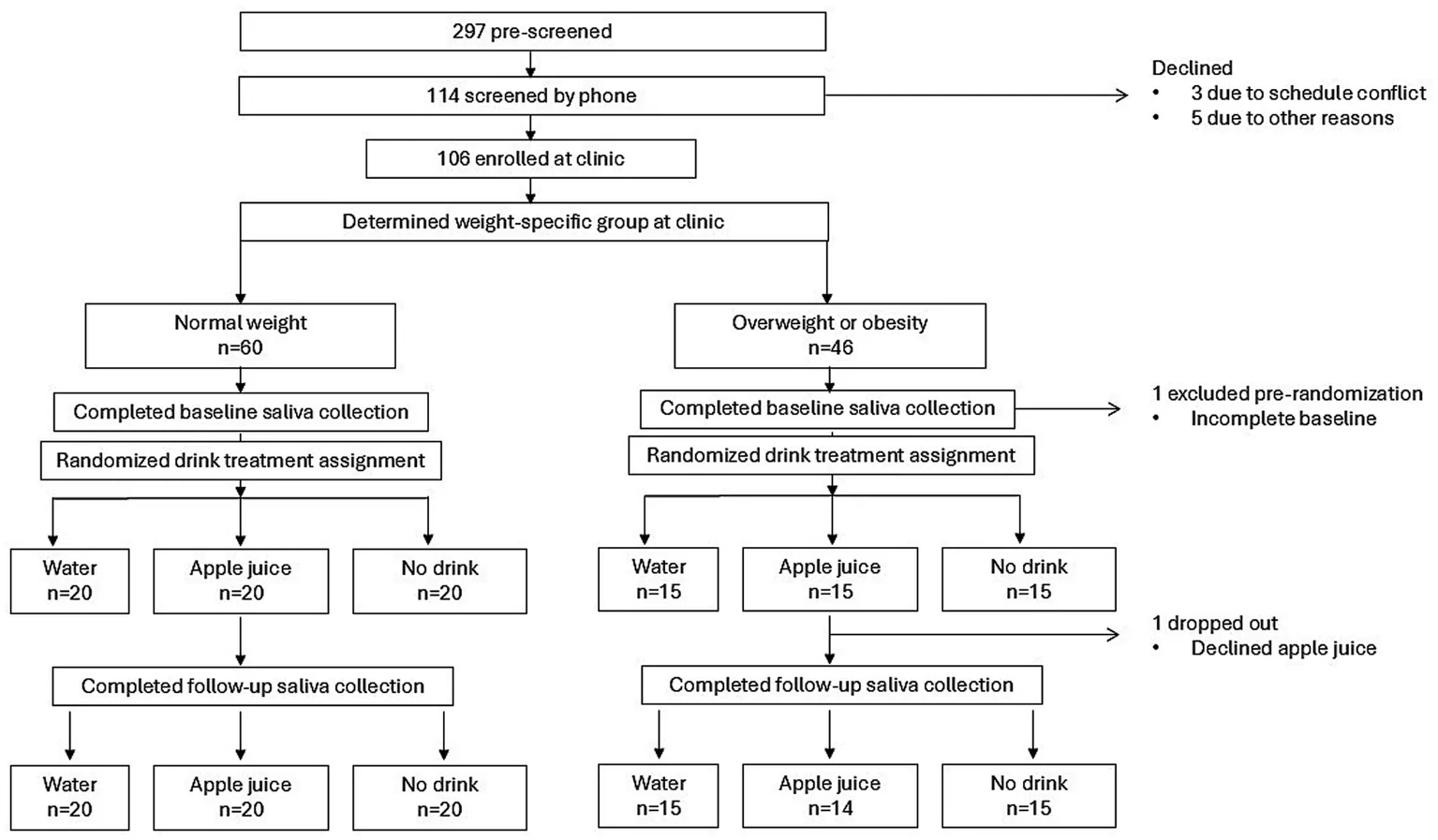
CONSORT flow diagram for the saliva insulin as biomarker randomized controlled trial.

### 2.3 Randomization

Randomization was stratified by weight status to account for potential effect modification due to higher insulin levels associated with overweight or obesity (34). Within each weight-specific group, participants had an equal chance of assignment to each of the three drink treatments. Prior to the start of the SIB RCT, a research assistant (MG), who was not involved in participant recruitment or analysis, prepared ordered, sealed envelopes, each containing a random assignment to one of 3 drink treatment groups. One set of envelopes was prepared for children with normal weight. One set was prepared for children with overweight or obesity. For each set of envelopes, a list of numbers (each from 1 to 60) was randomly scrambled using randomizer.org. Keeping the numbers in randomized order, MG put each number into an envelope and sealed the envelope. The sealed envelopes were then consecutively numbered on the outside of the envelope from 1 to 60. When the SIB RCT started, in consecutive order of enrollment, after each participant finished collecting their baseline saliva, the study coordinator (RC) opened one envelope to find out the randomly assigned drink treatment (Numbers 1–20: AJ; 21–40: W; 41–60: ND). No changes were made to the scrambled numbers or treatment assignment after trial commencement.

### 2.4 Drink treatments

Study participants assigned to the W group were given a standard serving of tap water, which includes fluoride (35). In San Francisco schools, a standard water bottle holds about 500 mL (16–18 oz) (36, 37). Study participants assigned to the AJ group were given a volume and type of apple juice aligned with USDA nutrition standards and Child and Adult Food Program guidelines, which allow for 8 oz. servings of 100% fruit juice in school meals (38). The AJ group received a commercially available 200 mL juice box containing 100% real apple juice, made from concentrate, with no sugar added and no artificial flavor. The apple juice contained 94 kcal, 23 g carbohydrate, 0.4 g protein, 2.1 mg vitamin C, 8.0 mg calcium, 9.2 mg magnesium, 215 mg potassium, 11.0 mg of phosphorus, and 18 mg sodium. The apple juice had a pH of 3.5 (39) and an osmolality of 754 mmol/kg, measured in triplicate, by study staff using a freezing point depression osmometer. Study participants assigned to the ND group received no drink. The glass of water or juice box were served at room temperature, with nothing else. Study participants consumed the drinks within 5 min.

### 2.5 Saliva collection and analysis

Baseline and follow-up saliva samples were collected by the passive drool method and stored frozen at −80 degrees Celsius in cryogenic tubes, within 2 h after collection. Saliva insulin was determined by Salimetrics lab (Carlsbad, CA, USA) and expressed as a dichotomous variable relative to 170 pg/mL (about 5 mU/L), a threshold value in other studies (40, 41). Saliva osmolality was determined, in triplicate, by freezing point depression osmometer and expressed relative to the same cutoff of 70 mmol/kg used in the prior analysis (27). Saliva osmolality is a hydration biomarker (42, 43).

Saliva metabolite abundance was determined by the University of California Davis, West Coast Metabolomics Center (WCMC) in 5 batches on consecutive days in January 2024. Primary metabolites were analyzed by untargeted GC-TOF MS using the WCMC validated automated liner exchange (ALEX) system with a Gerstel cold injection system (CIS 4) coupled to an Agilent gas chromatograph and a LECO Pegasus time-of-flight mass spectrometer. Samples were extracted and sequentially derivatized by methoximation and trimethylsilylation prior to analysis. Mass spectra were acquired under 70 eV electron ionization across a 50–650 m/z scan range. Raw data processing, peak deconvolution, and metabolite annotation were performed using the WCMC Primary Metabolism Database workflow (44–46). Abundance was expressed in terms of peak height (mass-to-charge ratio, m/z) and peak retention times (rt; in seconds), with known features labeled with their Kyoto Encyclopedia of Genes and Genomes (KEGG) ID, for all features that were positively detected in at least 10% of the samples.

### 2.6 Study questionnaire

Each study participant’s parent or guardian completed a questionnaire on behalf of the child, with input from the child. The study questionnaire captured information about factors that could confound effects of drinking water on insulin. The questionnaire asked about the date, time, quality and quantity of the last food consumed, last drinks other than water consumed, and last vitamins, gum, mint, cough drops, antacid or medicine consumed. Following NHANES survey methods, participants were considered fasted if they had not consumed anything other than drinking water for 8 h or more before study participation (47). To support inferences for school-based intervention planning, caregivers were asked if the study participant attended a public or private school.

The questionnaire asked when the study participant last consumed drinking water to index acute hydration condition. The questionnaire asked about the usual frequency of drinking water, modifying a question from Onufrak et al. (48): ‘In the past month, about how often did your child drink a glass or bottle of plain water? Include tap, water fountain, bottled, and unflavored sparkling water.’

To index the usual background consumption of caloric beverages, the questionnaire included validated (49) questions about beverage frequency on an average day, which are used by the Women Infants & Children (WIC) program in Los Angeles, California, in English, Spanish and Chinese (50). The questionnaire asked “on a usual day, about how many times does your child drink 100% juice?” next to a space to write in the “number of times per day”. The Caregivers were asked to report how many days their child was physically active for at least 60 min per day, a key indicator for physical activity (51, 52).

With respect to chronic health conditions, the caregiver and child were asked if a doctor or other health professional had ever told them that the child has diabetes, and if a doctor or health professional had ever told them that the child has asthma. The response options for the chronic health questions were: Yes, No, Borderline or pre-, Do not want to say, and Do not know.

### 2.7 Data analysis

All subjects were included in intention-to-treat analyses. Missing follow-up data for one person with overweight or obesity, who was assigned to the AJ group, but declined to drink apple juice and dropped out of the study, were replaced with the mean follow-up data for people of similar baseline condition (overweight or obesity), who consumed no drink. The W group served as reference in all analyses. For consistency with prior analyses (27), StataSE 15 software was used for logistic regression analyses. Python software was used to format metabolomic datasets, address zero feature abundance, and calculate within-person change in feature abundance. MetaboAnalyst.com 6.0 and R software were used for batch-error correction, data visualization, and pathway analyses.

#### 2.7.1 Baseline characteristics

The drink treatment groups were compared with respect to baseline characteristics. Weight-stratified logistic regression models tested for differences in the odds of ever-diagnosis of diabetes or asthma, weekly physical activity, usual daily juice intake, intake of drinking water within the past 24 h, arrival at the clinic after an 8 h fast, and baseline saliva insulin above 170 pg/mL.

#### 2.7.2 Saliva insulin and osmolality

Weight-specific logistic regression models tested if the W group had greater relative odds of saliva insulin below 170 pg/mL at follow-up than the AJ and ND groups. Weight-specific logistic regression models also tested if W resulted in significantly greater relative odds of saliva osmolality below 70 mmol/kg compared to AJ and ND. The models controlled for baseline level if the baseline level varied by drink treatment. The models, otherwise, did not control for covariates, given that the drink treatments were randomized. Logistic regression results with a *p*-value below 0.05 were considered statistically significant.

#### 2.7.3 Metabolomic profile

The metabolomic dataset received from the WCMC included a total of 362 features, including 137 known metabolites with KEGG IDs, per saliva sample. To correct for batch-related variation, raw feature abundance data were normalized using the EigenMS algorithm available in the MetaboAnalyst 6.0 platform (53–55). EigenMS was selected as the batch-correction method because, using MetaboAnalyst’s inter-batch distance metric, it produced a lower between-batch distance than ComBat normalization or the uncorrected data.

The abundance data were uploaded to MetaboAnalyst 6.0, log_2_ transformed and Pareto-scaled to visually describe the overall mean abundance of each/all feature/s for each drink treatment group, at baseline and follow-up, in heatmaps.

Using python software (IDLE 3.14.5), for each study participant, for each metabolite feature, within-person change in abundance was calculated as the absolute ratio of the follow-up value divided by the baseline value. After batch correction, before log transformation and/or calculation of change ratios, all data points were offset by 1 to account for zero abundance in 1539 out of 76,382 (2%) of features. The batch-error corrected, offset, within-person change ratios were subset for weight-specific pairwise comparison of AJ vs. W and ND vs. W groups.

For each data subset, using R software, the bottom 10% of features with lowest variance were filtered out, leaving 325 features in the analysis. For each weight-specific drink treatment group, the group mean within-person change ratio was calculated. Known and unknown metabolite features were classified as increased (mean AJ/W ≥ 1.5 or mean ND/W ≥ 1.5) or decreased (mean AJ/W ≤ 0.67 and mean ND/W ≤ 0.67) in the AJ and ND groups. The threshold cutoffs of 1.5 or 0.67 were chosen to optimize hypothesis generation about metabolic pathways potentially impacted. Biologically relevant changes can be masked by stricter filtering in hypergeometric pathway mapping (56, 57).

Using R software, for each weight-specific group, Orthogonal Partial Least Squares Discriminant Analysis (OPLS-DA) and Permutational Multivariate Analysis of Variance (PERMANOVA) were used to test the SIB RCT secondary hypotheses that (1) change in the overall metabolomic pattern in the AJ group differed significantly from change in the overall metabolomic pattern in the W group; and (2) change in the overall metabolomic pattern in the ND group differed from change in the overall metabolomic pattern in the W group. Drink treatment effect differences were operationalized as the log_2_ group mean within-person fold change ratio in the AJ group minus the log_2_ group mean within-person fold change ratio in the W group, and the log_2_ group mean within-person fold change ratio in the ND group minus the log_2_ group mean within-person fold change ratio in the W group. The OPLS-DA used the ropls package in R, one predictive component, and an optimized number of orthogonal components, selected by maximizing Q^2^ and checking for overfitting using 1,000 permutation tests. Results from the OPLS-DA were visualized with 95% confidence ellipses. Drink treatment effects were considered statistically significant if the OPLS-DA 95% confidence intervals did not overlap (58) and the OPLS-DA model permutation significantly discriminated between the treatment groups (Q^2^ *p*-value <0.05). The PERMANOVA used the R vegan package to test for significant differences in ellipse centroids based on Euclidean distances with 9,999 permutations. Drink treatment effects were considered significant in the PERMANOVA if the permutation-based *p*-value was below 0.05.

#### 2.7.4 Metabolic pathways potentially impacted

To identify metabolic pathways potentially impacted by the difference in drink treatment, KEGG IDs for known features that changed differently by 1.5-fold or more were uploaded to the Pathways module of MetaboAnalyst 6.0, with the *Homo sapiens* library. Features that decreased were entered separately from features that increased to facilitate interpretation about the direction of potential change in the pathways. Hypergeometric test *p*-values and pathway impact factors were used to evaluate if the listed features were overrepresented relative to what would be expected for the pathway by chance. Pathway impact factors add up the centrality and enrichment measures of each matched metabolite and then divide by the sum of the measures of all metabolites in the pathway. Pathways were considered potentially impacted if the raw *p*-value was <0.05 or the pathway impact factor exceeded 0.20. The FDR was calculated but not used to exclude potentially impacted pathways from hypothesis generation.

## 3 Results

### 3.1 Study participant characteristics

All participants in the SIB RCT were between the ages of 5 and 10 years. The participants self-reported Asian (52%), Hispanic (30%), Black or African American (5%), Multiracial (8%), or White (5%) race-ethnicity. All had Denti-Cal insurance, a proxy for low-income, and 91% attended public school. The weight-specific AJ and ND groups did not differ significantly from the W group, with respect to age, sex, race-ethnicity, chronic health condition, usual daily intake of 100% fruit juice, exercise for at least 300 min in the week before the study, intake of 3 or 4 glasses of drinking water within 24 h before study participation, intake of drinking water within 2 h of study participation, fasting status on arrival at the clinic, or baseline saliva insulin above 170 pg/mL (see Table 1). The only between-group difference noted, at baseline, was a significantly higher likelihood of saliva osmolality below 70 mmol/kg in the AJ vs. W group, in the children with normal weight.

**Table 1:** Study participant characteristics at baseline by weight status and randomly assigned drink treatment.

Weight statusAssigned drink treatment^a^	Normal weight					Overweight or obesity				
	Water	Apple juice		No drink		Water	Apple juice		No drink	
	%	%	*p*-value	%	*p*-value	%	%	*p*-value	%	*p*-value
Age 5 to 7 years	65	65	1.000	50	0.339	40	73	0.072	53	0.466
Male sex	60	55	0.749	70	0.508	47	47	1.000	47	1.000
Asian race-ethnicity	80	75	0.705	70	0.468	27	47	0.260	40	0.441
Hispanic race-ethnicity	10	20	0.384	10	1.000	67	40	0.148	53	0.458
Chronic health condition	5	5	1.000	15	0.314	20	13	0.626	13	0.626
Usual daily 100% fruit juice	35	40	0.744	35	1.000	60	27	0.072	40	0.277
Active at least 300 min last week^b^	50	40	0.526	45	0.752	53	53	1.000	40	0.466
3 or 4 glasses of water yesterday	40	40	1.000	65	0.117	47	60	0.466	33	0.458
8-h fasted before participation	5	10	0.556	11	0.526	20	14	0.685	13	0.626
Drank water within 2 h of the study	32	35	0.821	39	0.422	46	36	0.582	40	0.743
Saliva insulin >170 pg/mL	55	60	0.749	70	0.330	67	80	0.413	93	0.097
Saliva osmolality < 70 mmol/kg	55	20	0.027*	40	0.344	20	40	0.239	33	0.413

^a^Participants were randomly assigned to the Water, Apple juice, or No drink treatment groups. ^b^Active for at least 60 min per day on 5 or more days last week. *Value differs significantly from the corresponding value for the Water group (*p* < 0.05).

### 3.2 Weight-specific drink treatment effects on saliva insulin

At baseline, weight status modified the distribution of saliva insulin (see Figure 2). For children with normal weight, baseline saliva insulin ranged from 6 to 690 pg/mL, with a median of 208 pg/mL. For children with overweight or obesity, baseline insulin ranged from 28 to 5,070, pg/mL with a median of 422 pg/mL. Median saliva insulin was 214 pg/mL lower for children with normal weight.

**Figure 2:**
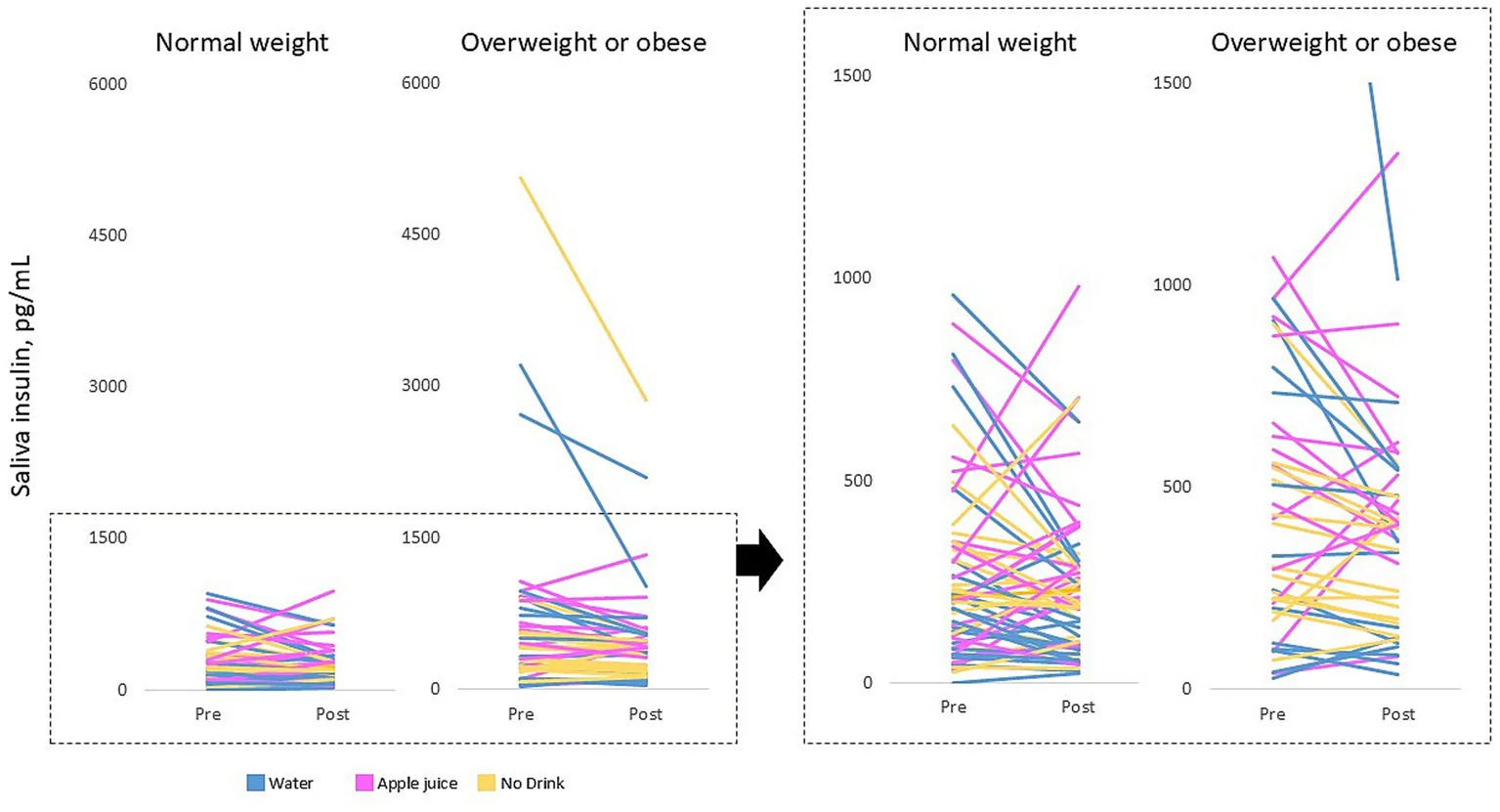
Saliva insulin before and 45–60 min after administration of the randomly assigned drink treatment for each study participant by weight status. Randomly assigned drink treatments were 500 mL tap water, 200 mL apple juice or no drink. The dotted box on the right zooms in to show greater detail for saliva insulin results in the 0–1,500 pg/mL range. The threshold for elevated saliva insulin in this study (170 pg/mL) corresponds to approximately 5 mU/L.

At follow-up, regardless of weight status, the majority of children who were given AJ had a saliva insulin of 170 pg/mL or higher (85% of children with normal weight; 87% of children with overweight or obesity). Also, regardless of weight status, the majority of children who were given ND had a saliva insulin of 170 pg/mL or higher (70 and 80%, respectively).

The effects of W were weaker by about half in the group with overweight or obesity. At follow-up, saliva insulin was below 170 pg/mL for 75% of children with normal weight and 47% of children with overweight or obesity. The weight-specific odds ratios for having saliva insulin below 170 pg/mL after W instead of AJ were 17.0 (95%CI: 3.5–83.4, *p*-value = <0.001) for children with normal weight and 5.7 (95%CI: 0.9–34.5, *p*-value = 0.059) for children with overweight or obesity. The weight-specific relative odds of lower saliva insulin after W instead of ND were 7.0 (95%CI: 1.7–28.2, *p*-value = 0.006) in children with normal weight and 1.6 (95%CI: 0.2–11.5, *p*-value = 0.626) in children with overweight or obesity. The relative odds of lower saliva insulin after W instead of either AJ or ND was 10 times greater in children with normal weight, and 4 times greater in children with overweight or obesity (OR, 95%CI: 10.3, 2.9–36.2, *p*-value<0.001 vs. 4.4, 1.1–17.7, *p*-value = 0.038).

### 3.3 Weight-specific drink treatment effects on saliva osmolality

Baseline saliva osmolality ranged from 49 to 204 mmol/kg, with a median of 74 mmol/kg, in the group with normal weight. It ranged from 56 to 163 mmol/kg, with a median of 79 mmol/kg, in the group with overweight or obesity. The normal weight group had a 38% higher relative odds of saliva osmolality below 70 mmol/kg and an 86% higher relative odds of osmolality below 100 mmol/kg, compared to the group with overweight or obesity, though the estimated differences were not statistically significant (OR, 95%CI: 1.38, 0.61–3.12, *p* = 0.444; 1.86, 0.67–5.17, *p* = 0.236). At baseline, 13% of the group with normal weight and 22% of the group with overweight or obesity had saliva osmolality over 100 mmol/kg.

Controlling for baseline differences in the group with normal weight, the relative odds of having a saliva osmolality below 70 mmol/kg at follow-up were significantly higher for the W group compared to the ND group (OR, 95%CI: 4.83, 1.05–22.09, *p* = 0.042). The estimated effect for W vs. AJ was not statistically significant (OR, 95%CI: 2.60, 0.58–11.60, *p* = 0.212). At follow-up, saliva osmolality was below 70 mmol/kg for 80%, 50%, and 45% of the W, AJ and ND groups, respectively.

In the group with overweight or obesity, follow-up saliva osmolality was below 70 mmol/kg for 53%, 80%, and 33% of the W, AJ, and ND groups, respectively. The unadjusted odds ratios for osmolality below 70 mmol/kg at follow-up were 0.29 (95%CI: 0.06–1.45, *p* = 0.130) for W vs. AJ and 2.29 (95%CI: 0.52–10.01, *p* = 0.273) for W vs. ND.

### 3.4 Weight-specific drink treatment effects on saliva metabolomic profile

Figure 3 summarizes results from weight-specific OPLS-DA at baseline and follow-up. At baseline, the saliva metabolomic profiles for the AJ and ND groups did not differ significantly from the profile for the W group. At baseline, the 95% confidence ellipses for the metabolomic profiles overlapped and the permutation and PERMANOVA tests were not statistically significant.

**Figure 3:**
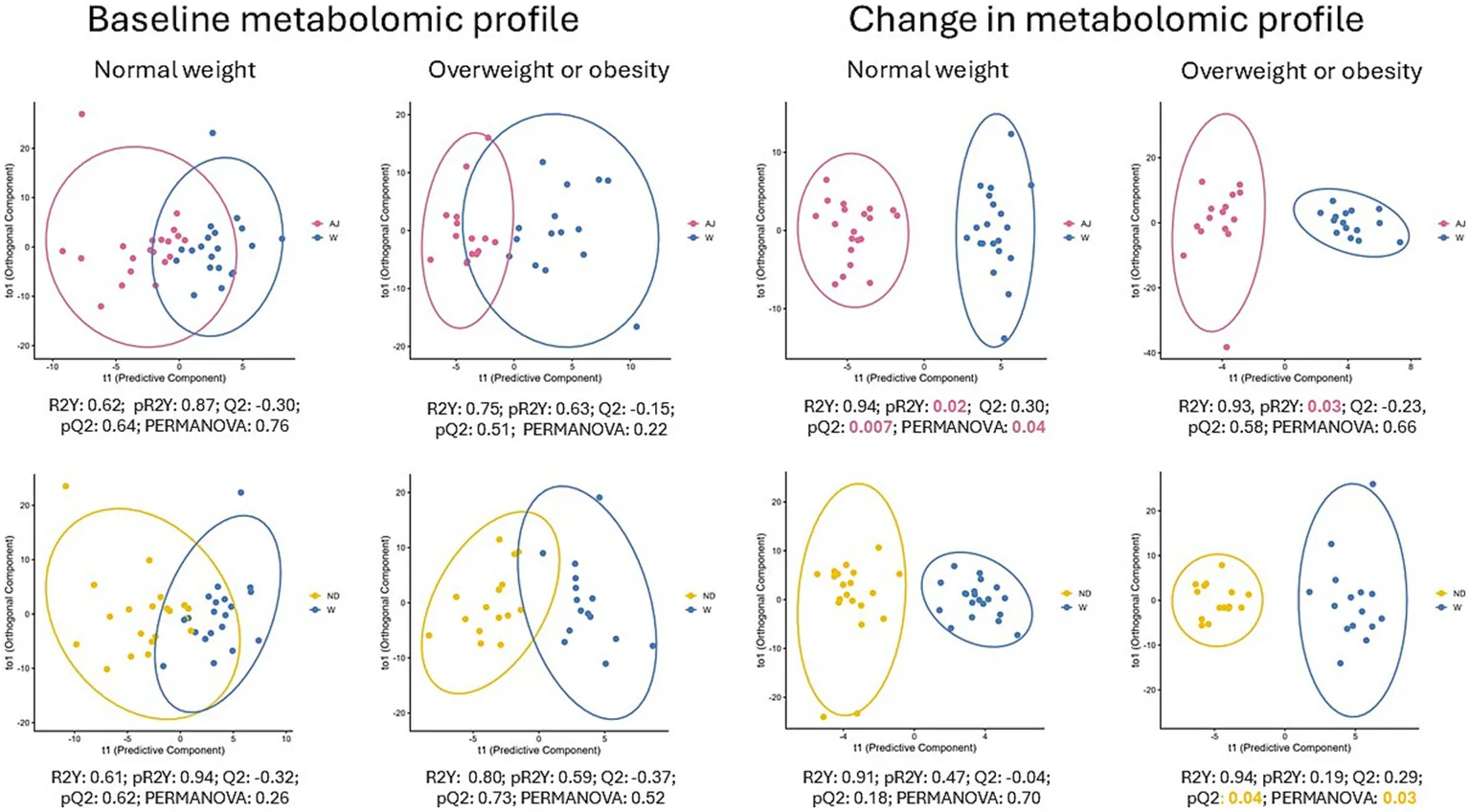
Saliva metabolomic profile at baseline and 45–60 min after the randomly assigned drink treatment by study participant weight status. Figures represent results from Orthogonal Partial Least Squares-Discriminant Analysis (OPLS-DA) with 95% confidence ellipses. The blue color highlights results for study participants assigned to drink water (W). The pink color represents study participants assigned to drink apple juice (AJ). The yellow color represents participants assigned to have no drink (ND).

After the randomly assigned drink treatment, the 95% confidence ellipses for the changes in saliva metabolomic profile did not overlap. In PERMANOVA, drinking water (W) had a statistically significant effect on change in overall saliva metabolomic profile, when compared to AJ in children with normal weight, and when compared to ND in children with overweight or obesity. The significant OPLS-DA models explained over 90% of the between-group variance (AJ vs. W in NWT: R^2^Y = 0.94; ND vs. W in OWT: R^2^Y = 0.94) with modest but stable predictive performance (AJ vs. W in NWT: Q^2^ = 0.30, *p* = 0.007; ND vs. W in OWT: Q^2^ = 0.29, *p* = 0.04).

The heatmaps in Figure 4 (left) summarize the saliva feature abundance in the weight-specific groups before and after the AJ, ND, and W treatments. The scatterplots in Figure 4 (right) describe the direction and log transformed magnitude of each change in feature abundance in the AJ and ND groups, relative to the corresponding change in the W group. In the group with normal weight, after AJ, 86 features increased and 46 features decreased by 1.5-fold or more; After ND, 111 features increased and 61 features decreased by 1.5-fold or more. In the group with overweight or obesity, 57 features increased and 69 features decreased, after AJ; After ND, 56 features increased and 62 features decreased. The fold changes of all of the features detected, known and unknown, are provided in the Appendix Tables 1a–h. Table 2 names the known features (compounds with KEGG ids) that increased or decreased by 1.5-fold or more.

**Figure 4:**
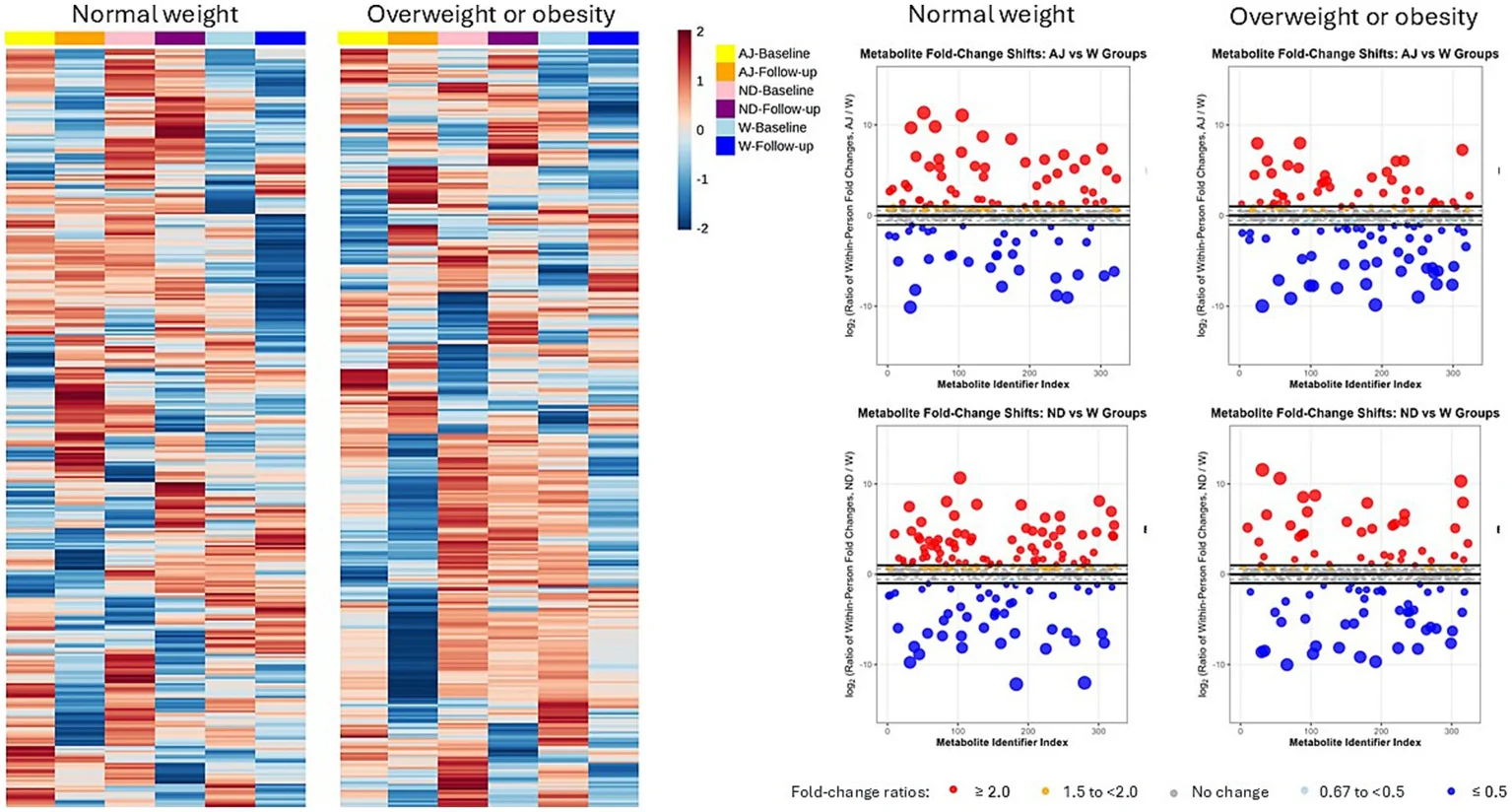
Change in saliva metabolite abundance after apple juice or no drink instead of drinking water by study participant weight status. Legend: The heatmap (left) summarizes the average absolute change in saliva abundance of each metabolite (y-axis, metabolite labels not shown) for each weight specific drink treatment group. Each of four scatterplots, shown on the right, summarizes the log ratio of the mean change in saliva abundance (y-axis) of each metabolite (x-axis) in each weight-specific group. The ratios for the mean change in the group assigned to have apple juice (AJ) and the group assigned to have no drink (ND) were calculated relative to the mean change in saliva abundance observed in the corresponding weight-specific group assigned to drink water (W). In the scatterplots, a 2-fold change is equal to log2FC = 1.

**Table 2:** Mean within-person fold change in saliva feature abundance in the apple juice and no drink groups relative to the water group by weight status.

Metabolite (mz/rt)	KEGG Id	BinBase name	Normal weight		Overweight or obesity	
			AJ vs. W	ND vs. W	AJ vs. W	ND vs. W
192/537804	C00079	phenylalanine	0.003	0.004		
174/546741	C00986	1,3-diaminopropane	0.019	0.235	0.365	
133/455754	C00872	aminomalonic acid	0.029	0.038		
262/531892	C06771	triethanolamine	0.030	0.016	0.265	
361/976828	D04845	maltitol	0.036	0.479	0.007	
156/600315	C00064	glutamine	0.045	0.048		
319/657941	C00221	glucose	0.049	0.326	255.685	372.258
226/623789	C00043	UDP-N-acetylglucosamine	0.199	0.237		
173/553450	C00312	xylulose	0.219	0.191		
361/946601	C00208	maltose	0.290	0.011	1.729	1570.604
174/211668	C00022	pyruvic acid	0.304			1.703
204/569637	C22350	levoglucosan	0.326		4.316	0.123
257/611345	C00438	N-carbamoylaspartate	0.380	0.317		
204/932179	C01971	cellobiose	0.444	0.082	0.045	4.806
202/780482	C00078	tryptophan	0.647		0.157	0.257
160/433227	C00049	aspartate	1.561	2.457		
305/730022	C00137	myo-inositol	1.571	1.765		
213/1095668	C03845	zymostenol	1.575	1.571		0.615
315/845976	C03546	inositol-4-monophosphate	1.576	1.667		
217/633603	C07326	1,5-anhydroglucitol	1.607	1.962	0.004	0.004
191/278829	C01089	3-hydroxybutyric acid	1.611	0.419		
241/385816	C00106	uracil	1.650	21.956		35.692
117/573663	C08352	6-deoxyglucose	1.660		1.541	
314/345365	C00009	phosphate	1.670			
247/506306	C02630	2-hydroxyglutaric acid	1.679	0.016	0.592	
292/497572	C01620	threonic acid	1.809			0.574
236/918039	C00212	adenosine	1.829	1.570	11.574	
217/594647	C00103	glucose-1-phosphate	1.847	1.579		
292/489385	C00639	isothreonic acid	1.929			
245/390775	C00122	fumaric acid	1.937		0.036	0.033
261/421596	C00489	glutaric acid	1.950			
441/730691	C00366	uric acid	1.973			
158/548028	C01042	N-acetylaspartic acid	2.081	5.179		
217/573587	C00532	lyxitol	2.430	1.638		0.669
217/467595	C16884	threitol	2.706		1.913	
174/594051	C00077	ornithine	3.176	0.002		
339/779120	C00712	oleic acid	3.272	14.589	25.296	1.734
254/586317	C00295	orotic acid	3.296	17.604	0.618	0.528
193/620200	C00230	3,4-dihydroxybenzoic acid	3.403	0.161	8.782	
304/488730	C00334	4-aminobutyric acid	3.482	1.966		
315/804750	C05345	fructose-6-phosphate	5.302	89.939		1.795
103/544100	C00181	xylose	6.348		2.425	
387/818275	C00092	glucose-6-phosphate	7.289	12.061	38.247	21.163
267/699866	C00785	urocanic acid	7.633	1.505	0.501	
345/632897	C00296	quinic acid	8.472	1.831	2.777	0.502
217/667922	C00794	sorbitol	10.929		252.310	
89/310761	C00233	2-ketoisocaproic acid	19.487			
104/434422	C05629	hydrocinnamic acid	19.897	5.032	0.002	1.692
265/619128	C00262	hypoxanthine	40.561	3.093		
233/463180	C00711	malic acid	42.448	13.075	2.538	0.026
202/684929	C00954	indole-3-acetate	75.354	14.474	46.738	42.139
164/368081	C07086	phenylacetic acid	91.683	0.504	65.190	95.920
273/617342	C00158	citric acid	126.991		0.005	0.002
179/598147	C01744	3-(4-hydroxyphenyl)propionic acid	424.831			
142/364716	C00148	proline	825.463			3014.802
191/219848	C01432	lactic acid	896.448	10.895	1.742	0.001
129/1078536	C00187	cholesterol	2140.058	1625.031	1.602	
246/913814	C00270	N-acetylneuraminic acid	2605.832	1.578		
299/604335	C00346	phosphoethanolamine			0.172	0.003
117/205778	C03046	butane-2,3-diol		0.004	0.005	0.004
243/507619	C02277	dodecanol		23.254	0.591	0.205
315/1038688	C00020	adenosine-5-monophosphate		12.680	0.307	0.415
189/326574	C00086	urea				0.545
160/578299	C02095	fucose		10.534		1.548
180/366992	C00253	nicotinic acid		55.195	2.141	1.571
248/455267	C05145	3-aminoisobutyric acid				1.619
217/567437	C00379	xylitol		0.201	0.263	1.708
174/588872	C00134	putrescine		179.227		2.080
227/610734	C00597	3-phosphoglycerate		213.328	14.226	2.190
156/404121	C00408	pipecolinic acid		2.231		3.833
217/553135	C00121	ribose		1.677		11.878
189/377495	C00258	glyceric acid		265.169	0.296	17.454
319/648756	C00984	galactose		1.709	4.234	22.378
307/639442	C02336	fructose		7.419	0.534	119.825
157/621404	C00327	citrulline			0.634	
255/420133	C00178	thymine			1.514	
177/278969	C0108	34,135			1.549	
311/704511	C08362	palmitoleic acid			1.966	
230/897434	C00294	inosine		7.356	2.602	
337/777414	C01595	linoleic acid		8.658	4.419	
176/483560	C00073	methionine		3.799	5.625	
259/723539	C00499	allantoic acid			6.152	
179/542795	C00642	4-hydroxyphenylacetic acid			21.984	
271/915139	C00089	sucrose		2.976	22.240	
174/623532	C02037	glycyl-glycine		0.616		
132/978597	C08320	lignoceric acid		1.502		
117/920648	C08281	behenic acid		1.543		
371/901749	C01885	1-monopalmitin		1.770		
154/664395	C00135	histidine		1.796		
357/593299	C03189	glycerol-alpha-phosphate		2.148		
247/370608	C00042	succinic acid		2.372		
308/654036	C03672	p-hydroxylphenyllactic acid		2.688		
203/959214	D01947	1-monostearin		3.057		
191/948197	C01083	trehalose		3.453		
204/998425	C01235	galactinol		3.483		
264/646534	C00147	adenine		16.372		
317/610551	C08261	azelaic acid		21.745		

m/z/rt, Mass-to-charge ratio; rt, retention time; KEGG id, Kyoto Encyclopedia of Genes and Genomes identifier; Changes in metabolomic features were measured in saliva before and after having apple juice (AJ) or no drink (ND) and expressed as a ratio, relative to the same features’ change in saliva induced by drinking water (W). All features listed in the table changed by 1.5-fold or more compared to the change following drinking water. Each value in the table is a group mean within-person ratio for the change in saliva metabolic feature abundance after AJ or ND, relative to the mean within-person change for that metabolic feature in the W group. Ratios that increased are highlighted in red. Ratios that decreased are highlighted in blue.

### 3.5 Metabolic pathways potentially impacted by drink treatment

Table 3 describes the metabolic pathways potentially impacted by the 1.5-fold or greater changes in known compounds. The Venn diagram in Figure 5 summarizes the overlap in potential metabolic impacts across the study treatments and weight-specific groups.

**Table 3:** Metabolic pathways potentially impacted by apple juice or no drink compared to drinking water by study participant weight status.

Drink treatment/Reference	Direction of change	Metabolic pathway	Total	Expected	Hits	Raw p-value	FDR	Impact
Normal weight								
Apple Juice/Water	Decreased	Alanine, aspartate and glutamate metabolism	28	0.17588	3	0.000539	0.043122	0.11378
		Glycolysis or Gluconeogenesis	26	0.16332	2	0.010653	0.39861	0.09806
		Glyoxylate and dicarboxylate metabolism	32	0.20101	2	0.015933	0.39861	0
		Nitrogen metabolism	6	0.037688	1	0.037159	0.49545	0
		Phenylalanine metabolism	8	0.050251	1	0.049266	0.56304	0.35714
		Phenylalanine, tyrosine and tryptophan biosynthesis	4	0.025126	1	0.024913	0.39861	0.5
		Pyrimidine metabolism	39	0.24497	2	0.02325	0.39861	0.02495
	Increased	Alanine, aspartate and glutamate metabolism	28	0.52764	5	0.000122	0.009758	0.39904
		Arginine and proline metabolism	36	0.67839	3	0.028362	0.32414	0.22906
		Arginine biosynthesis	14	0.26382	3	0.001913	0.062942	0.06383
		Butanoate metabolism	15	0.28266	3	0.00236	0.062942	0.03175
		Galactose metabolism	27	0.50879	3	0.013023	0.20837	0.00995
		Histidine metabolism	16	0.30151	2	0.034976	0.33196	0.12295
		Inositol phosphate metabolism	30	0.56533	3	0.017401	0.23201	0.15918
		Neomycin, kanamycin and gentamicin biosynthesis	2	0.037688	1	0.037345	0.33196	0
		Pentose and glucuronate interconversions	19	0.35804	3	0.004777	0.095533	0.07229
		Starch and sucrose metabolism	18	0.3392	2	0.04357	0.34856	0.27337
No Drink/Water	Decreased	Alanine, aspartate and glutamate metabolism	28	0.22864	2	0.020648	0.33037	0.11378
		Arginine biosynthesis	14	0.11432	2	0.005302	0.1624	0.06383
		Butanoate metabolism	15	0.12249	2	0.00609	0.1624	0
		Nitrogen metabolism	6	0.048995	1	0.04808	0.4808	0
		Pentose and glucuronate interconversions	19	0.15515	2	0.009737	0.19473	0.16867
		Phenylalanine metabolism	8	0.065327	2	0.001677	0.13419	0.35714
		Phenylalanine, tyrosine and tryptophan biosynthesis	4	0.032663	1	0.032295	0.43061	0.5
		Pyrimidine metabolism	39	0.31847	2	0.038478	0.43975	0.02495
No Drink/Water	Increased	Alanine, aspartate and glutamate metabolism	28	0.61558	4	0.002758	0.073539	0.39664
		Arginine and proline metabolism	36	0.79146	2	0.18631	0.74523	0.23488
		beta-Alanine metabolism	21	0.46168	3	0.009874	0.15798	0
		Butanoate metabolism	15	0.32977	2	0.041218	0.33793	0.03175
		Galactose metabolism	27	0.59359	5	0.000218	0.017454	0.13748
		Glycerolipid metabolism	16	0.35176	2	0.046466	0.33793	0.30374
		Histidine metabolism	16	0.35176	3	0.004483	0.089666	0.34426
		Inositol phosphate metabolism	30	0.65955	3	0.02633	0.30091	0.15918
		Linoleic acid metabolism	5	0.10992	1	0.10532	0.51701	1
		Neomycin, kanamycin and gentamicin biosynthesis	2	0.04397	1	0.0435	0.33793	0
		Nicotinate and nicotinamide metabolism	15	0.32977	2	0.041218	0.33793	0
		Purine metabolism	70	1.5389	5	0.016512	0.22016	0.10503
		Starch and sucrose metabolism	18	0.39573	4	0.000482	0.019286	0.3236
Overweight or obesity								
Apple Juice/Water	Decreased	Alanine, aspartate and glutamate metabolism	28	0.21106	2	0.017662	0.45581	0.0024
		Arginine biosynthesis	14	0.10553	2	0.004509	0.36075	0.23936
		Citrate cycle (TCA cycle)	20	0.15075	2	0.009181	0.36724	0.12019
		Glyoxylate and dicarboxylate metabolism	32	0.24121	2	0.02279	0.45581	0.14
	Increased	Galactose metabolism	27	0.32224	3	0.00352	0.14082	0.09176
		Linoleic acid metabolism	5	0.059673	1	0.058337	0.76369	1
		Neomycin, kanamycin and gentamicin biosynthesis	2	0.023869	1	0.023734	0.63292	0
		One carbon pool by folate	26	0.3103	2	0.036993	0.73986	0.05077
		Purine metabolism	70	0.83543	3	0.047588	0.76141	0.00416
		Starch and sucrose metabolism	18	0.21482	3	0.001051	0.084114	0.2618
No Drink/Water	Decreased	Alanine, aspartate and glutamate metabolism	28	0.15829	2	0.009954	0.26543	0.0024
		Arginine biosynthesis	14	0.079146	2	0.002497	0.19977	0
		Citrate cycle (TCA cycle)	20	0.11307	2	0.005122	0.2049	0.12019
	Increased	Arginine and proline metabolism	36	0.36181	3	0.004861	0.38886	0.20465
		Glycine, serine and threonine metabolism	33	0.33166	2	0.041717	0.47677	0.02475
		Glycolysis or Gluconeogenesis	26	0.26131	2	0.02675	0.42799	0.09806
		Glyoxylate and dicarboxylate metabolism	32	0.32161	2	0.039418	0.47677	0.06
		Neomycin, kanamycin and gentamicin biosynthesis	2	0.020101	1	0.020006	0.42384	0
		Pentose phosphate pathway	23	0.23116	2	0.021192	0.42384	0
		Starch and sucrose metabolism	18	0.1809	2	0.013197	0.42384	0.21157

**Figure 5:**
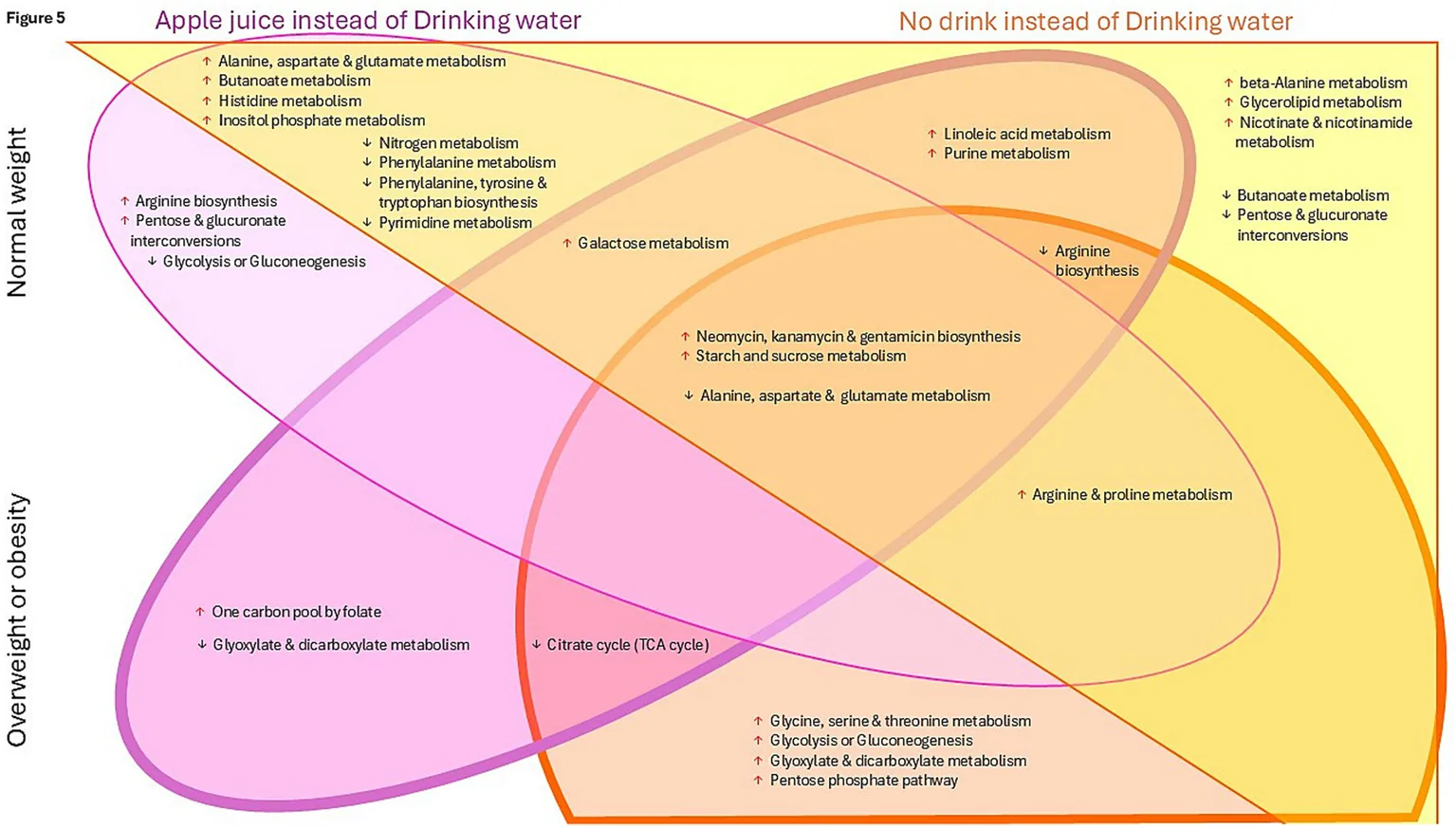
Venn diagram summarizing metabolic pathways potentially impacted by the randomly assigned drink treatments by study participant weight status. Legend: Metabolic pathways potentially impacted by 200 mL apple juice are highlighted by pink color. Metabolic pathways potentially impacted by no drink are highlighted in yellow orange. The darker colors represent potential effects in the group with overweight or obesity.

#### 3.5.1 Weight-independent drink treatment effects

Regardless of participant weight status, for both AJ vs. W and ND vs. W, potential metabolic impacts of metabolites that increased included starch and sucrose metabolism and neomycin, kanamycin & gentamicin biosynthesis (i.e., potential impact on oral microbiome activity, given that humans do not synthesize antibiotics). Metabolites that decreased in abundance potentially impacted alanine, aspartate, and glutamate metabolism.

Regardless of weight status, AJ vs. W was associated with potential increases in galactose metabolism. Regardless of weight status, after ND vs. W, metabolites of arginine and proline metabolis appeared to increase; metabolites involved in arginine biosynthesis appeared to decrease.

#### 3.5.2 Normal weight-specific drink treatment effects

Pathways potentially impacted by both AJ vs. W and ND vs. W, but only in the group with normal weight, included increased intermediates of alanine, aspartate and glutamate metabolism, butanoate metabolism, histidine metabolism, and inositol phosphate metabolism, and decreased metabolites of nitrogen metabolism, phenylalanine metabolism, phenylalanine, tyrosine and tryptophan biosynthesis, and pyrimidine metabolism.

Potential impacts of AJ vs. W, observed only in the group with normal weight, were increased intermediates of arginine biosynthesis and pentose and glucuronate interconversions, and decreased intermediates of glycolysis or gluconeogenesis. Potential impacts of ND vs. W, observed only in the group with normal weight, included increased abundance of metabolites involved in beta-alanine metabolism, glycerolipid metabolism and nicotinate and nicotinamide metabolism, and decreased intermediates of butanoate metabolism and pentose and glucuronate interconversions.

#### 3.5.3 Overweight or obesity-specific drink treatment effects

In the group with overweight or obesity, both AJ vs. W and ND vs. W potentially decreased citrate cycle (TCA cycle) activity. After AJ vs. W, only in the group with overweight or obesity, there was increased abundance of intermediates of one carbon pool by folate and decreased abundance of metabolites involved in glyoxylate and dicarboxylate metabolism. Potential impacts of ND vs. W, included increased intermediates of glycine, serine and threonine metabolism, glycolysis or gluconeogenesis, glyoxylate and dicarboxylate metabolism, and pentose phosphate pathway activity.

## 4 Discussion

In the SIB RCT, randomly assigned drink treatments induced significantly different acute changes in saliva insulin in healthy, non-acutely ill, children (27). Saliva insulin was significantly more likely to be normalized within 1 h after 500 mL drinking water (W) instead of one serving of apple juice (AJ) or no drink (ND). The present analysis weight-stratified the SIB RCT drink treatment effects on saliva insulin and tested for corresponding AJ vs. W and ND vs. W effects on saliva metabolomic profile. Metabolic pathways potentially impacted by the drink treatments were described, recognizing that different pathways might be impacted, if insulin is elevated for different reasons (e.g., greater insulin secretion after AJ vs. less insulin clearance after ND). The SIB RCT randomization was weight-stratified in case of effect modification by weight status. The underlying goal of the SIB RCT was to gather causal data that might inform decisions about school-based drinking water intervention design, dose, process measures, and target outcomes.

### 4.1 SIB RCT hypotheses

#### 4.1.1 Expectations about insulin

The SIB RCT hypothesized drink treatment effects on metabolomic profile, given observed drink treatment effects on insulin, because acute changes in insulin have well-established effects on metabolism. Classical postprandial effects of insulin are to lower blood glucose by promoting glucose uptake, utilization, and storage, while suppressing endogenous glucose production, protein breakdown, lipolysis, and use of fat as a metabolic fuel (59, 60). Insulin stimulates uptake of glucose and amino acids into insulin-sensitive tissues, increases glycolysis and protein synthesis in muscle (61), and inhibits gluconeogenesis and glycogenolysis in the liver (59, 60). Excess glucose results in lipogenesis and triglyceride synthesis in the liver (62, 63).

Blood insulin normally increases above fasting levels within 2 to 5 min after carbohydrates, protein, or amino acids enter the bloodstream (64–66). The increase in blood insulin peaks between 30 and 60 min and returns to fasting levels, below 10 uIU/mL (about 350 pg/mL), in 2 to 3 h, as the liver and kidneys clear insulin from the blood (64–66). In healthy individuals, the maximum acute blood insulin response after 50 g carbohydrate is about 20–30 uIU/mL (64, 66).

Saliva insulin tracks with blood insulin levels following both high- and low-carbohydrate meals and is significantly higher with overweight or obesity in adults and children (31, 41). In the present study, after AJ, saliva insulin was 7 times higher in the group with overweight or obesity (5,070 vs. 690 pg/mL). In insulin resistant individuals, the response to a 75 g oral glucose load is known to be markedly exaggerated and prolonged, with peak blood insulin concentrations that can exceed 150–200 uIU/mL and total insulin exposure (area under the curve) several-fold greater than in insulin-sensitive individuals (23, 67).

#### 4.1.2 Expected weight-specific effects

The SIB RCT anticipated weight-specific effects on metabolomic profile because obesity-related insulin resistance is characterized by altered metabolism, kinase activation, mitochondrial dysfunction, and nutrient congested or stalled Kreb cycle (21, 68). Activated kinases, including protein kinase C (PKC), trigger serine or threonine phosphorylation of the insulin receptor substrate (IRS), rather than the normal tyrosine phosphorylation of IRS (21, 69), which dampens intracellular insulin responses, such as the signaling cascade that normally synthesizes and uses inositol phosphate second messengers (70). Obesity and insulin resistance are associated with a distinct metabolomic signature, which includes uninhibited protein breakdown, impaired catabolism of branched chain amino acids (BCAA) (71–73), unsuppressed lipolysis, mitochondrial overload, increased lactate, altered redox balance, and increased α-Hydroxybutyrate (α-HB), a biomarker of oxidative stress (74). The significantly altered metabolomic profile has been observed in the saliva of children with obesity (75).

#### 4.1.3 Expected effects of No drink

ND effects on metabolomic profile were hypothesized because, besides carbohydrate intake and insulin resistance, as drivers of elevated insulin, impaired insulin sensitivity due to extracellular hyperosmolality (76), i.e., underhydration (77), can also increase insulin. Hyperosmotic cell shrinkage in the liver promotes protein breakdown, gluconeogenesis and glycogenolysis, whereas hypo-osmotic cell swelling counteracts proteolysis and glycogen breakdown (19, 78). In healthy, young men, experimentally induced decreases in plasma osmolality from 286+/−1 to 265+/−1 mosm/kg, achieved by intravenous administration of desmopressin, liberal water drinking and infusion of hypotonic saline, decreases plasma insulin, total body leucine flux (a proxy for whole-body protein breakdown), and whole-body leucine oxidation rate (a proxy for irreversible loss of amino acids), and increases glucose metabolic clearance rate (during hyperinsulinaemic-euglycemic clamping), plasma non-esterified fatty acids, and lipid oxidation (78, 79).

### 4.2 SIB RCT findings

As hypothesized, the present weight-stratified analyses found significant effects of AJ vs. W and ND vs. W on acute changes in saliva metabolomic profile, which corresponded with significant drink treatment effects on saliva insulin. Contrary to expectation, however, the significant effect of AJ vs. W on overall metabolomic profile was only observed in the normal weight group. The significant effect of ND vs. W was only observed in the group with overweight or obesity. Consistent with warnings in the literature (6, 17, 26), weight status did appear to modify the effects tested in this study, resulting in null effects for some groups.

The weaker effect of AJ vs. W on saliva metabolomic profile in the group with overweight or obesity mirrored weaker effects of AJ vs. W on saliva insulin in that group. The stronger effect of ND vs. W on metabolomic profile in the group with overweight or obesity co-occurred with weaker effects of ND vs. W on saliva osmolality in that group. After W, in the group with overweight or obesity, the proportion with saliva insulin below 170 pg/mL was about half that observed in the group with normal weight (47% vs. 75%). After W, in the group with overweight or obesity, a greater proportion remained relatively hypertonic (47% vs. 20% remaining hypertonic in the group with normal weight). Further research is needed to consider baseline hydration condition as a potential effect modifier and determine whether the dose of W can be tailored for significant effect of AJ vs. W in groups with overweight or obesity and significant effect of ND vs. W in groups with normal weight.

Consistent with similar but muted effects, after AJ vs. W in the group with overweight or obesity, and after ND vs. W in the group with normal weight, the drink treatments appeared to potentially impact similar metabolic pathways, regardless of weight status, but reach different levels of statistical significance (see Appendix Figures 1, 2). After AJ vs. W, for example, intermediates of alanine, aspartate, and glutamate metabolism decreased in both the group with normal weight and the group with overweight or obesity. Both the raw *p*-value and FDR for effect were below the 0.05 criterion for statistical significance in the group with normal weight, but only the raw *p*-value was statistically significant in the group with overweight or obesity. After ND vs. W, intermediates of starch and sucrose metabolism increased, with significant *p*-value<0.0005 and FDR < 0.05 in the group with normal weight, but with *p*-value<0.013 and non-significant FDR = 0.42 in the group with overweight or obesity.

The overall metabolomic effects and potential metabolic impacts, common across treatments and study groups, in this study suggest that drinking water interventions might be hypothesized to significantly impact intermediates of alanine, aspartate and glutamate metabolism and starch and sucrose metabolism, in all children, regardless of weight condition – assuming intervention participants are like the present study population. If the interventions are designed to test these two specific metabolic pathways, with targeted assays and methods that index pathway flux, if multiple testing can be avoided, a raw *p*-value might suffice to test for intervention effectiveness.

As all children who participated in this study had Denti-Cal insurance, a proxy for low income, the hypothesized effects can be expected to apply to socioeconomically disadvantaged groups, at higher risk of chronic health conditions, including caries and obesity (28, 29). In San Francisco, to reduce local health disparities, drinking water interventions aim to reach and benefit precisely this group of children via school-based interventions open to all children in public schools. As this study does not include higher income children, at lower health risk, the results may not generalize to all children in all schools. Intervention planners can stratify evaluation design by income to align with this study.

### 4.3 Hypothesis generation for the future

The SIB RCT was designed to test a-priori hypotheses about change in overall untargeted metabolomic profile and describe changes in profile in terms of changes in relative metabolite abundance and potential metabolic impacts. Although the weight-stratified design was not well-powered to single out individual metabolite biomarkers or metabolic pathways, potentially impacted by the drink treatments, the descriptive SIB RCT data may be useful for hypothesis generation about metabolites or pathways to consider, in future, as possible process measures or target outcomes for drinking water intervention. Each of the pathways summarized in Figure 5 might be hypothesized to respond significantly to AJ vs. W and/or ND vs. W intervention, in children with normal weight and/or overweight or obesity. Based on Figure 5, however, most of the metabolic impacts would only be expected for a particular intervention, AJ or ND, and/or population, with or without overweight/obesity.

#### 4.3.1 Effects potentially mediated or modified by insulin sensitivity and the TCA cycle

Potential pathway impacts, observed in the group with normal weight, but not in the group with overweight or obesity, suggest hypotheses about drink treatment effects that depend on insulin sensitivity and efficiently running TCA cycle. After AJ vs. W and ND vs. W, in the group with normal weight, only, the saliva abundance of intermediates of inositol phosphate metabolism (glucose-6-phosphate, D-myo-inositol-4-phosphate, and myo-inositol) increased. Inositol phosphates are critical intracellular second messengers in the insulin receptor signaling cascade. The pathway is known to be impaired in obesity (70).

Also, after AJ vs. W and ND vs. W, and only in the group with normal weight, the saliva abundance of some metabolites involved in alanine, aspartate, and glutamate metabolism *increased* (Appendix Figure 1). The observed increases imply increased TCA cycle activity for the group with normal weight, because increased L-aspartic acid can convert to oxaloacetate, which synthesizes citric acid by accepting acetyl-CoA, which initiates the TCA cycle. In the group with overweight or obesity, intermediates of TCA cycle activity *decreased* by 1.5-fold or more after AJ vs. W and ND vs. W.

#### 4.3.2 Effects potentially mediated by hypertonicity

Potential pathway impacts, observed after ND vs. W, suggest hypotheses about underhydration- or hypertonicity-mediated drink treatment effects on endogenous carbohydrate metabolism. Unlike AJ which contains 3 grams of sucrose per 8 ounces (80), ND provides no nutrients. Increased intermediates of starch and sucrose metabolism, observed after both AJ vs. W and ND vs. W, therefore, imply effects of both AJ vs. W and ND vs. W on *endogenous* carbohydrate metabolism. In the group with normal weight, after both AJ vs. W and ND vs. W, the saliva abundance of glucose-1-phosphate, a marker of gluconeogenesis (81), increased by over 1.5-fold compared to the changes after W (see Appendix Figure 2). The potential impact of ND vs. W on glycine, serine and threonine metabolism, in the group with overweight or obesity, would be consistent with ND vs. W increasing the production of precursors for gluconeogenesis (82, 83). An increase in gluconeogenesis after AJ, in healthy normal weight individuals, is not consistent with the expected classical effect of insulin to suppress gluconeogenesis. The AJ vs. W exposure, *which is evaluated relative to water*, may mix effects of relative hypertonicity with classical effects of carbohydrate.

The ND treatment, which provided no water, conceivably created or sustained relative hypertonic conditions, at least locally, in the mouth. AJ, with an osmolality of over two times greater than the osmolality of blood (754 mmol/kg vs. 285 mmol/kg) was hypertonic relative to W. After both AJ vs. W and ND vs. W, the saliva abundance of myo-inositol, sorbitol and/or alpha-d-galactose, which participate in protective responses to osmotic stress and cell volume regulation (84–86) increased by 1.5-fold or more. After ND vs. W, the observed increase in saliva abundance of trehalose (Appendix Figure 2) suggests that, 1 h after the drink treatment, oral bacteria had activated protective osmolytes to defend cell volume against osmotic stress (87, 88). Hypertonic stress shifts metabolism to increase osmolyte concentrations to defend cell volume (89). In the mouth, oral bacteria (e.g., Streptococcus and Lactobacillus species) sense the host’s hypertonic saliva. They absorb the overflowing glucose, activate their OtsA/OtsB enzyme pathways, and synthesize trehalose to protect their cell walls from collapsing (90).

A decreased saliva abundance of metabolites involved in alanine, aspartate, and glutamate metabolism, after ND vs. W, suggests hypotheses about hypertonicity-mediated drink treatment effects on protein metabolism, anaplerosis to sustain the TCA cycle, and amino acid-derived endogenous glucose production. Hypertonic stress regulates protein metabolism (91). Alanine, aspartate and glutamate are pulled out of circulation to convert to Krebs cycle intermediates to sustain the TCA cycle if/when mitochondrial processing is compromised (92, 93). Cells release less alanine when the TCA cycle is disrupted (94). Decreased saliva abundance of glutamine might reflect use of glutamine as a substrate for gluconeogenesis, which is increased by hypertonicity (79). In postabsorptive states, glutamine is a primary substrate for gluconeogenesis in the kidneys and intestine (95).

#### 4.3.3 Effects potentially mediated by the pentose phosphate pathway

Potential pathway impacts observed in the group with overweight or obesity suggest hypotheses about metabolic targets involved in the one carbon pool, pentose phosphate pathway, and galactose metabolism. Children with overweight or obesity might be hypothesized to respond to AJ vs. W with increases in one carbon pool by folate and galactose metabolism. Children with overweight or obesity might be hypothesized to respond to ND vs. W with increased flux through the pentose phosphate pathway. Increased galactose metabolism allows the cell to bypass rate-limiting glycolytic checkpoints and divert carbohydrate away from (overloaded) mitochondria to glycogen storage or glycoprotein synthesis (96). Galactose metabolism is upregulated in response to osmotic stress (96). Increased flux through the pentose phosphate pathway generates NADPH, which favors fatty acid synthesis and gluconeogenesis (97), and is characteristic of Warburg-like metabolism (98).

#### 4.3.4 Potential impacts on fat metabolism

Observed potential impacts on linoleic acid metabolism, beta-alanine metabolism, and glycerolipid metabolism suggest hypotheses about fat metabolism after AJ vs. W in children with overweight or obesity and after ND vs. W in children with normal weight. Linoleic acid is an essential dietary fatty acid that can be incorporated into lipids, elongated/desaturated into other ω-6 fatty acids, or oxidized for energy. Increased linoleic acid pathway activity could theoretically reflect greater fat synthesis and/or greater fat oxidation or altered utilization. Greater fat synthesis may be more likely than greater fat oxidation, after ND or AJ, because, in humans, hyperosmotic stress suppresses mitochondrial fatty acid oxidation, inducing Warburg-like metabolic remodeling that prioritizes aerobic glycolysis (burning glucose without oxygen) (99). After AJ vs. W, in children with overweight or obesity, increased fat synthesis might further be hypothesized to reduce osmotic stress related to the juice composition by reducing solute concentrations, by converting hydrophilic solute to hydrophobic lipid (effectively removing it from solution) (100, 101). After ND vs. W, in children with normal weight, if the TCA cycle is *not* impaired, given fasting/normalized insulin levels, then increased fat oxidation might also be expected, to mitigate osmotic stress via production of metabolic water. Fat oxidation produces more metabolic water than carbohydrate oxidation (102).

Increased beta-alanine metabolism and glycerolipid metabolism can be hypothesized to favor fat synthesis after ND vs. W in children with normal weight. Beta-alanine metabolism produces malonyl-CoA, a key substrate for fatty acid synthesis. Glycerolipid metabolism generates glycerol-3-phosphate, the backbone for the triglyceride molecule. Glycerolipid metabolism can be hypothesized to protect against ND-related hyperosmotic conditions, via glycerol, an important osmolyte (103). The finding of increased glycerolipid metabolism after ND vs. W, in the group with normal weight but not the group with overweight or obesity, might suggest hypotheses about effect mediation by glycerolipid/FFA futile cycling, which is reduced in obesity (104).

#### 4.3.5 Potential effects on the oral microbiome

Pathways potentially impacted in this analysis suggest hypotheses about AJ vs. W and ND vs. W effects on oral microbiome. Regardless of weight status, after both AJ vs. W and ND vs. W, this untargeted metabolomics analysis detected relative increases in the saliva abundance of aminoglycosides, which are not produced by humans, but are produced by bacteria (105). One hour after ingestion of fructose, glucose and sucrose from AJ, the potential impact on neomycin, kanamycin and gentamicin biosynthesis could reflect a spike in oral bacterial fermentation of sugar (106, 107). After ND, the result could be hypothesized to reflect more bacteria in the mouth, compared to after W (rinsed mouth).

In the group with normal weight, the analysis also observed relative increases in intermediates of butanoate metabolism, a sign of anaerobic bacterial conversion of lactate to produce butyrate (108, 109). In the group with normal weight, the saliva abundance of lactic acid increased about 900-fold after AJ vs. W. The lack of corresponding effect in the group with overweight or obesity is consistent with microbial dysbiosis in obesity, including reduced capacity of the microbiome to generate butyrate (110).

#### 4.3.6 Potential mechanisms of effects of drinking water

The results of this study suggest potential for drinking water to reduce metabolic risk factors for chronic disease via insulin-related mechanisms. Acute insulin-mediated effects would, of course, need to be replicated and hold true for longer than 1 h. Insulin-related mechanisms would add to a list of proposed or hypothesized causal mechanisms linking drinking water or hydration with reduced risk of chronic disease, including, for example, other hormones (vasopressin, aldosterone, cortisol), inflammation, oxidative stress, and cell volume change (19, 111–121).

With respect to mechanisms of effect, importantly, the results of this study, like other studies of drinking water or hydration on overall metabolomic profile (122–124), signal potential for drinking water to have *many* metabolic impacts, at the same time. On the plus side, co-occurring effects might imply opportunity for efficient public health intervention against multiple outcomes simultaneously. Alternatively, multiple causal mechanisms, all in play together, might mean that complicated interactions can modify effects of interest. Further research is needed to elucidate hydration effects on metabolism.

#### 4.3.7 Health implications of drink treatment effects

The potential short-term effects observed in this study suggest hypotheses about longer-term health implications. Although the results pertain only to a very short window of time, 45–60 min after the drink treatment, the results pertain to drink treatments that are frequently available options for children during the school day. If the short-term effects repeat, every time the drink treatment happens, if the effects are not canceled out by compensatory adaptations, and if the effects accumulate over time, then daily metabolic impacts might be expected to gradually impact the risk of obesity and chronic health problems. Further research is needed to test longer-term effects.

In the above analysis and discussion, effects of AJ and ND were presented relative to W. Readers need to flip the reference condition to specify hypotheses about the potential beneficial impact(s) of drinking water intervention, i.e., W vs. AJ or W vs. ND.

### 4.4 Limitations

Limitations of the SIB RCT are discussed elsewhere, including parallel group, instead of crossover, design, participant eligibility restricted to health network patients able to access only one particular dental clinic, eligibility unrestricted by hydration condition or caries risk, results pertaining only to the specific drink treatments tested, short 45–60 min follow-up, between-treatment group differences at baseline, despite randomization, and missing follow-up data for one out of 105 participants (27). Despite randomization, between-group differences related to unobserved factors, such as toothbrushing or flossing before study participation, could also be a source of error.

With regard to the present metabolomic analysis, the randomized weight-specific treatment groups did not differ significantly at baseline with respect to saliva insulin level or metabolomic profile, and missing follow-up data did not appear to alter the direction or significance of the results. Although limited statistical power, due to the small sample sizes in the weight-stratified groups, might conceivably explain why the groups did not differ significantly at baseline, the sample size provided adequate power to detect statistically significant differences at 45–60 min for AJ vs. W in the group with normal weight and ND vs. W in the group with overweight or obesity. Statistical vulnerabilities of OPLS-DA might conceivably account for the statistically significant between-group differences at follow-up (58), but OPLS-DA with the same drink treatment factors specified, at baseline, did not produce significant differences. The study involved balanced design, very low sparsity (0.8% zeros overall) and minimal group bias in zeros (AJ 1.0% vs. W 0.6%; ND vs. W).

The results of this study are restricted in focus on metabolomic profiles *in saliva*. Biomarkers in saliva can reflect an individual’s physiological status or susceptibility to systemic disease, but metabolite concentrations may be lower in saliva and/or different from metabolic features in other types of specimens (123, 125, 126). Saliva has limitations as a proxy of whole-body metabolism. Processes, such as amylase action to digest starch in the mouth, may be relevant in saliva, but not other parts of the body. To the extent that saliva secretion rate and composition are controlled by autonomic and sympathetic nerves, tightly regulated, and modulated by medications and hormones, saliva metabolites may not be perfect indices of metabolic pathway activity (127, 128). This analysis did not control for saliva creatinine because creatinine is not filtered at a stable rate into saliva like it is into urine, factors unrelated to systemic hydration determine salivary creatinine, and control for changes due to change in salivary flow rate might mask hydration-related effects of interest (129).

Metabolomic results reflect the global, downstream, combined product of genetics, proteomics, microbiota, age, gender, environment, stressors, behaviors including diet and activity, oral and systemic diseases, hormones and medications (125, 126, 130). While the present saliva metabolomic results reflect more than mere activity of metabolic pathways, they are not all encompassing. The untargeted metabolomics results of this study assess hydrophilic metabolism and include low molecular weight metabolites, carbohydrates and sugar phosphates, amino acids, hydroxyl acids, free fatty acids, purines, pyrimidines, aromatics, and exposome-derived chemicals. This analysis did not assess hydrophobic metabolites, such as ceramides, sphingomyelins, cholesteryl esters, lyso- and phospholipids, mono-, di- and triacylglycerols, galactosyl- and glucuronyllipids, involved in cell membrane biology, signaling, and energy storage (130). Between-batch differences in WCMC metabolomic assays are a potential source of error in this analysis.

The metabolomic data in this study were expressed in terms of retention index and mass peak heights, not in terms of absolute concentrations. Future studies should add internal standards and use calibration curves to determine absolute (micromolar) concentrations for TCA (Krebs cycle) intermediates, glycolysis, pentose phosphate pathway, or other metabolites.

Despite the randomized study design, causal inference about drinking water impacts on metabolism is limited, because metabolomic analyses are not flux or pathway analyses. If a given metabolite is increased, it is not possible to determine, from the present dataset, if synthesis of the metabolite was increased, breakdown was decreased, or both. The present results suggest hypotheses to test to confirm and characterize drinking water effects on metabolic pathways. Given that painless, non-invasive saliva collection could enable more complete, population-representative monitoring of metabolic health and intervention planning, greater capacity to interpret saliva metabolomic profiles holds promise for public health.

The present analysis considered overweight and obesity as proxy for insulin resistance and fluid restriction (no drink) as proxy for underhydration. The study was not designed, however, to test effects of drinking water specifically mediated via insulin and/or hydration-specific mechanisms.

## 5 Conclusion

In the SIB RCT, an acute bolus of 500 mL drinking water (W) resulted in significantly different change in saliva metabolomic profile over 45–60 min, compared to 200 mL apple juice (AJ) for children with normal weight, and compared to no drink (ND) for children with overweight or obesity. The weight-specific effects on saliva metabolomic profile were consistent with observed weight-specific effects on saliva insulin. The differences in change in metabolomic profile reflected 1.5-fold or greater differences in hundreds of metabolites. Metabolic pathways potentially impacted in this study generated hypotheses about drinking water effects on the TCA cycle, pentose phosphate pathway, gluconeogenesis, amino acid metabolism, galactose metabolism, glycerolipid metabolism, and the oral microbiome. Given the qualitatively different potential metabolic impacts, in the descriptive data, by drink treatment (AJ vs. W or ND vs. W) and weight-specific group, future research can anticipate heterogeneous metabolic effects and possible effect modification. The results underscore need for drinking water interventions to carefully specify beverage change goal(s) and account for participant weight and baseline status.

In summary, this RCT addressed gaps in data for causal inference about drinking water effects on metabolism. The study reports causal effects of specific drink exposures (AJ vs. W and ND vs. W) on saliva metabolomic profile. The exposures tested align with beverage options available to children during the school day. Regarding ‘how much plain water’ to recommend or target for intervention, the data pertain to a single serving of 500 mL tap water at room temperature. Regarding if the water should be ‘consumed alone or instead of other beverages’, the results indicate potential for statistically significant impact, either if the water is consumed alone, and/or instead of one 200 mL serving of apple juice. The descriptive data from this RCT signal opportunity to track specific metabolites as process measures and/or target outcomes. Regarding ‘what magnitude of effect to expect’ drinking water intervention to have on saliva metabolites in children, though not tested for statistical significance, the relative fold-changes reported here may serve as preliminary data. Further research is warranted to characterize metabolomic effects of locally relevant drink options, not only in San Francisco, but for children in communities around the world. Drinking water interventions hold promise to significantly impact insulin-related metabolic endpoints.

## Data Availability

The datasets presented in this article are not readily available because a data use agreement is required. Requests to access the datasets should be directed to jodidstookey@gmail.com.
